# Detection of ARGs from Gram-Negative Bacteria in Positive Blood Cultures Using a Microarray-Based System: Towards a Molecular Antibiotic Susceptibility Assay

**DOI:** 10.3390/antibiotics14121221

**Published:** 2025-12-04

**Authors:** Cataldo Maria Mannavola, Giordana Cafaro, Barbara Fiori, Roberto Rosato, Francesca Romana Monzo, Tiziana D’Inzeo, Brunella Posteraro, Maurizio Sanguinetti, Flavio De Maio

**Affiliations:** 1 Department of Basic Biotechnological Sciences, Intensive and Perioperative Clinics, Università Cattolica del Sacro Cuore, 00168 Rome, Italy; cataldomaria.mannavola01@icatt.it (C.M.M.); tiziana.dinzeo@unicatt.it (T.D.); brunella.posteraro@unicatt.it (B.P.);; 2 Department of Laboratory and Hematological Sciences, Fondazione Policlinico Universitario A. Gemelli IRCCS, 00168 Rome, Italy; giordana.cafaro@gmail.com (G.C.); barbara.fiori@policlinicogemelli.it (B.F.); francescaromana.monzo@policlinicogemelli.it (F.R.M.); 3 Microbiota Analysis & Microbial WGS Research Core Facility, Gemelli Science and Technology Park (GSTeP), Fondazione Policlinico Universitario A. Gemelli IRCCS, 00168 Rome, Italy; roberto.rosato@unicatt.it; 4 Precision Medicine in Clinical Microbiology Unit, Direzione Scientifica, Fondazione Policlinico Universitario A. Gemelli IRCCS, 00168 Rome, Italy

**Keywords:** antimicrobial resistance, fast microbiology, DNA microarray, WGS

## Abstract

**Background/Objectives**: Antimicrobial resistance (AMR) represents a major global health challenge, driving the need for rapid and accurate diagnostic tools. Novel molecular assays, including multiplex PCR and DNA microarray-based systems, have emerged to detect antimicrobial resistance genes (ARGs) alongside bacterial identification. **Methods**: In this study, we evaluated the performance of the HybriSpot12 PCR AUTO (HS12a) system and the MDR Direct Flow Chip (MDR-FC) Kit—an automatic microarray assay based on reverse hybridization—for the detection of ARGs directly from positive blood culture (PBC) samples. A total of 111 Gram-negative bacterial isolates (92 Enterobacterales, 14 *Acinetobacter baumannii*, and 6 *Pseudomonas* spp.), previously characterized by whole-genome sequencing (WGS), were each used to generate a PBC, which was then analyzed with the HS12a/MDR-FC assay. **Results**: We demonstrated perfect agreement for the detection of macrolide resistance genes across all bacterial species and high agreement for genes conferring resistance to sulfonamides and β-lactams. In contrast, aminoglycoside resistance genes showed only moderate agreement, with minor discrepancies observed in *Klebsiella pneumoniae* and *Escherichia coli*, largely attributable to specific SNP variations. **Conclusions:** The HS12a/MDR-FC assay includes 51 ARGs, though not all were represented in our isolate set, and some false negatives were observed. Despite these limitations, its broad coverage and rapid turnaround remain advantageous compared to other rapid assays with fewer targets. Future refinements should aim at broader gene coverage, inclusion of key mutations, and detection of emerging variants, making this approach a promising tool for rapid AMR surveillance and antimicrobial stewardship.

## 1. Introduction

Antimicrobial resistance (AMR) is a major global challenge that requires urgent and continuously updated countermeasures [1]. In this context, antimicrobial susceptibility testing (AST) is rapidly evolving, with new assays designed to ensure accuracy, shorten turnaround times, and detect emerging antibiotic resistance genes (ARGs) [2,3]. The European Commission’s In Vitro Diagnostic Regulation (IVDR), in force since May 2022, has further emphasized the need for clinical microbiology laboratories to verify manufacturers claimed performance for AST assays [4].

Reducing the delay to appropriate antimicrobial therapy is crucial, given the wide range of potentially resistant bacteria [5,6,7]. A key driver of AMR dissemination is the plasmid-mediated horizontal transfer of ARGs [8], which can originate from diverse reservoirs (environment, animals, or humans). However, our understanding of the ecological and evolutionary dynamics that promote the emergence of these plasmids in clinical pathogens remains incomplete [9,10]. Plasmids can be exchanged across species and ecological niches under selective pressures (e.g., antibiotic exposure), acting as efficient vehicles for ARG mobilization. This dynamic has driven the global spread of clinically relevant determinants such as *bla*_CTX-M_, *bla*_KPC_, *bla*_NDM_, and *bla*_OXA-48-like_ [11]. These patterns strengthen the rationale for molecular assays that directly target resistance genes rather than inferring resistance from species identity or phenotype.

Several commercial assays are now available to detect common ARGs in clinically relevant bacterial species. However, most platforms are validated on a limited panel of high-prevalence species and a small set of ARG targets, so reported performance may not generalize to the broader diversity encountered in routine diagnostics. From this perspective, broad-spectrum, rapid approaches are essential to reduce diagnostic delays, minimize the risk of selecting multidrug-resistant (MDR) strains, and support antimicrobial stewardship [12]. Rapid molecular tests have been increasingly integrated into routine microbiology, allowing simultaneous pathogen identification and resistance gene detection. Widely adopted systems include the FilmArray Blood Culture Identification 2 Panel (BioFire; FA), the LAMP-based eazyplex^®^ BloodScreen GN (Amplex Diagnostics), and the Alifax Molecular Mouse (MM) [13,14,15]. Despite their utility, the coverage of these systems remains limited: FA detects 23 species and 7 ARGs, MM up to 35 species and 13 ARGs, and eazyplex LAMP-GN only 5 species and 2 ARGs. Mass spectrometry remains a reliable, cost-effective alternative [16]. Other systems (e.g., eazyplex^®^ SuperBug complete B, Novodiag^®^ CarbaR+, Amplidiag^®^ CarbaR+MCR) focus mainly on carbapenemase genes, detecting 6–11 targets [16]. Their turnaround times (TATs) range from 30 min for very rapid platforms (Novodiag, eazyplex) to ~1.5 h for FA, and up to 5 h for other systems [17]. In general, rapid microbiological technologies are defined by a time-to-result (TTR) ≤8 h, with many current systems achieving results within 1 to 5 h, thus enabling timely detection of ARGs and pathogen identification [18].

In this study, we evaluated the combined use of multiplex PCR (HybriSpot12 PCR AUTO; HS12a) with DNA microarray (MDR-FC Kit), allowing detection of more than 50 ARGs in a single assay. The HS12a/MDR-FC system, marketed by Eurospital Diagnostic (Trieste, Italy), is CE-IVD–approved and involves multiplex PCR amplification with biotinylated primers, automated reverse hybridization on a probe-coated membrane, and visualization through a colorimetric reaction. Images are captured by an integrated camera and interpreted by Hybrisoft software (https://emb.eurospital.com/, accessed on 30 November 2025), providing results within 4 h from different sample types, including rectal/nasal swabs, blood cultures, and bacterial colonies. For this evaluation, 111 Gram-negative bacterial isolates, previously characterized by whole-genome sequencing (WGS), were used to generate positive blood cultures. WGS data were reanalyzed in depth to investigate results lacking agreement (particularly false negatives and false positives), i.e., genes present in the genome but not detected by the assay or genes detected by assay but not revealed in the genomes.

## 2. Results and Discussion

We tested 111 Gram-negative bacterial organisms with matched WGS data (Supplementary Data S1) using the HS12a/MDR-FC assay on simulated bacterial positive blood cultures (PBCs). The complete ARG call-sets from WGS and HS12a/MDR-FC are reported in Supplementary Data S2 and S3, respectively.

**Figure 1:**
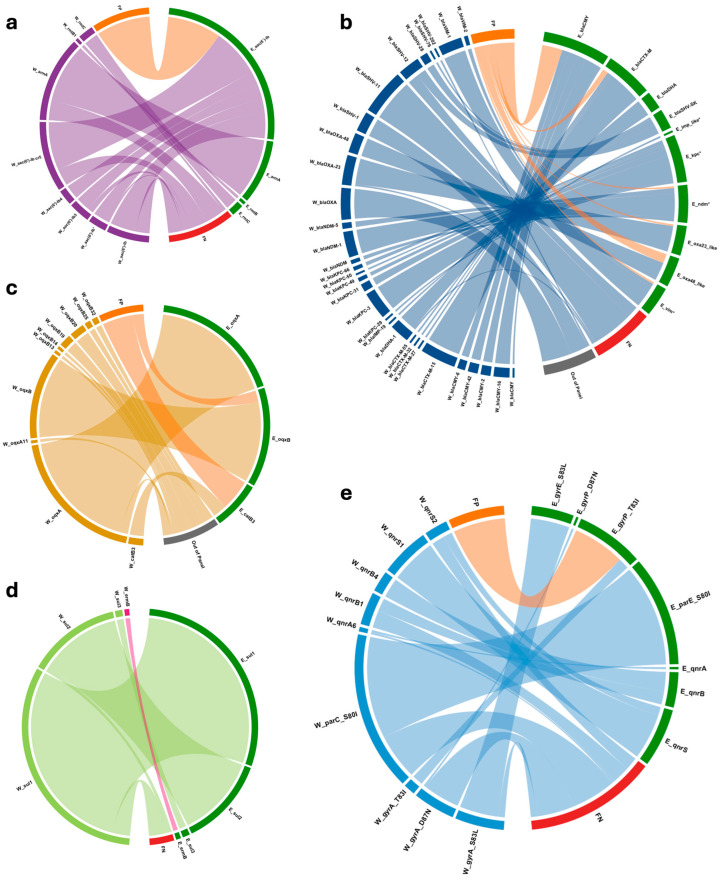
**Concordance of AMR gene detection by HS12a/MDR-FC and WGS.** Chord-chart plots showing the comparison between AMR gene detection results by HS12a/MDR-FC (Eurospital) and WGS assays. Results are stratified by (**a**) aminoglycosides (*aac*(6’)-*Ib*, *armA*, *rmtB*, *rmtC*), (**b**) β-lactams (*bla*_CMY_, *bla*_CTX-M_, *bla*_DHA_, *bla*_SHV-S_, *bla*_SHV-SK_, *bla*_IMP_-like, *bla*_KPC_, *bla*_NDM_, *bla*_OXA-23_-like, *bla_OXA-48_*-like, *bla*_VIM_), (**c**) phenicols/quinolones (*oqxA*, *oqxB*, *catB3*), (**d**) sulfonamides (*sul1*, *sul2*, *sul3*), and (**e**) quinolones (*gyrA* mutation points S83L, T83I and D87N and *parE-S80I*, *qnrA*, *qnrB*, *qnrS*). WGS-detected genes are shown with the prefix “W_”, while Eurospital-detected genes are indicated with the prefix “E_”. False positives: “FP”; false negatives: “FN”, “Out of panel” indicates genes not included in the HS12a/MDR-FC panel, “*” indicates which have multiple variants, as reported in the manufacturer’s instruction.

A global comparison between HS12a/MDR-FC and WGS results across antibiotic classes is summarized in Figure 1. The chord diagrams provide an overview of the distribution of detected ARGs, areas of agreement and instances of over-/under-detection between the two approaches. This graphical presentation illustrates, at a glance, the relative performance of the assay across aminoglycosides, β-lactams, macrolides, phenicols/quinolones, and sulfonamides, while also pinpointing instances of false-positive and false-negative results.

To facilitate a more detailed interpretation, the following sections present the results separately by antibiotic class, integrating numerical data (Table 1, Table 2 and Table 3) with focused discussion. This structure allows both a comprehensive assessment of the assay’s overall performance and a critical evaluation of gene- or species-specific patterns of agreement and non-agreement.

### 2.1. Detection of Genes Conferring Resistance to β-Lactams

The MDR-FC assay detected *bla*_CMY_, *bla*_CTX-M_, *bla*_DHA_, *bla*_SHV-S_, *bla*_SHV-SK_, *bla*_IMP_-like, *bla*_KPC_, *bla*_NDM_, *bla*_OXA-23_-like, *bla*_OXA-48_-like, and *bla*_VIM_ genes. A comparative analysis with WGS showed overall good agreement (Table 1; Figure 1b), with sensitivity (SE) of 0.73 (95% CI, 0.66–0.80) and specificity (SP) of 0.97 (95% CI, 0.96–0.98). The positive (PPV) and negative predictive (NPV) values were 0.82 (95% CI, 0.75–0.88) and 0.96 (95% CI, 0.94–0.97), respectively. These estimates indicate a low false-positive rate (SP = 0.97) and that some true positives were missed (SE = 0.73).

At the single-gene level, agreement was excellent for *bla*_CTX-M_ (SE = 1.00; SP = 0.96; PPV = 0.83; NPV = 1.00; McN test = 2.25; *p* = 0.13), *bla*_DHA_ (SE = 0.89; SP = 1.00; PPV = 1.00; NPV = 0.99; McN test = 0.00, *p* = 1.00), *bla*_NDM_ (SE = 0.94; SP = 0.98; PPV = 0.89; NPV = 0.99; McN test = 0.00, *p* = 1.00), *bla*_IMP_-like (SE = 1.00; SP = 1.00; PPV = 1.00; NPV = 1.00; McN test = Not applicable), and *bla*_OXA_ variants (*bla*_OXA23_-like: SE = 0.93; SP = 1.00; PPV = 1.00; NPV = 0.99; McN test = 0.00, *p* = 1.00; and *bla*_OXA48_-like: SE = 0.80; SP = 0.94; PPV = 0.57; NPV = 0.98; McN test = 1.12, *p* = 0.28) genes. Of the 21 *bla*_CTX-M_ genes correctly detected by the MDR-FC assay, WGS identified 18 as *bla*_CTX-M-15_; the remaining three were *bla*_CTX-M-27_ (n = 1), bla_CTX-M-32_ (n = 1), and *bla*_CTX-M-55_ (n = 1) (see Supplementary Data S2).

Relevant discrepancies were observed for *bla*_CMY_ (SE = 0.87; SP = 0.84; PPV = 0.59; NPV = 0.96; McN test = 58.82, *p* = 0.01) and especially for *bla*_SHV_ alleles (*bla*_SHV-S_: SE = 0.08; SP = 1.00; PPV = 1.00; NPV = 0.56; McN test = 45.02, *p* < 0.001; and *bla*_SHV-SK_: SE = 0.80; SP = 1.00; PPV = 1.00; NPV = 0.99; McN test = Not applicable). The manufacturer’s probes target two ESBL-associated motifs: *bla*_SHV-S_ (alleles carrying the G238S substitution; codon change GGC→AGC at nucleotide ~700 [19]) and *bla*_SHV-SK_ (alleles carrying both G238S and E240K substitutions; E240K corresponds to the codon change GAG→AAG at nucleotide ~703 [19]). Despite this design, several WGS-confirmed variants went undetected, including clinically relevant ESBL types such as *bla*_SHV-12_. Excluding the non-ESBL variants *bla*_SHV-1_ and *bla*_SHV-11_, WGS identified 5 *bla*_SHV-12_, 4 *bla*_SHV-28_, 1 *bla*_SHV-76_, and 1 *bla*_SHV-202_; only 4/5 *bla*_SHV-12_ were correctly detected by MDR-FC (see Supplementary Data S2). By design, *bla*_SHV-S_ is expected to recognize variants such as *bla*_SHV-2_ (G238S), whereas *bla*_SHV-SK_ encompasses variants such as *bla*_SHV-5_, *bla*_SHV-12_, and *bla*_SHV-15_ (G238S+E240K). Consequently, *bla*_SHV-11_ and *bla*_SHV-1_—both lacking G238S—are expected to test negative, which is consistent with our findings and with their non-ESBL status [20]. Moreover, *bla*_SHV-1_ remains biologically relevant as a likely progenitor of ESBL lineages, including *bla*_SHV-12_ [21,22]. To further contextualize under-detection, we examined representative, probe-region–proximal SNP/indel patterns from WGS (see Supplementary Data S4). Although the proprietary probe sequences are not publicly available, SNPs clustered around codons 238–240 in certain *bla*_SHV-12_ organisms plausibly introduce 3′-proximal mismatches that can impair hybridization, offering a mechanistic explanation for false negatives. Conversely, a *bla*_SHV-11_ organism (wild-type at 238/240) tested negative as expected given probe specificity. A complete map of *bla*_SHV_ alleles (*bla*_SHV_-1, -11, -12, -28, -76, -202) and their SNP/indel profiles is provided in Supplementary Data S4 and can inform future probe re-design/coverage expansion.

These findings are of clinical importance, as *bla*_SHV_ variants contribute to resistance to oxyimino-cephalosporins and, in some cases, ceftazidime/avibactam, impacting empirical therapy and stewardship strategies. Moreover, this under-detection translated into lower apparent prevalence in MDR-FC results when compared with the true genomic background.

Detection of carbapenemase genes such as *bla*_KPC_ (SE = 0.77; SP = 0.99; PPV = 0.96; NPV = 0.92; McN test = 3.12, *p* = 0.07) and *bla*_NDM_ was robust, although the assay was unable to resolve allelic variants (e.g., *bla*_KPC-31_), which may have direct therapeutic implications. Of the 37 *bla*_KPC_ genes identified by WGS—*bla_KPC_*_-3_ (n = 14), *bla_KPC_*_-29_ (n = 14), *bla_KPC_*_-31_ (n = 4), *bla_KPC_*_-49_ (n = 2), *bla_KPC_*_-50_ (n = 1), and *bla_KPC_*_-66_ (n = 2)—the MDR-FC assay correctly detected 24 (see Supplementary Data S2).

In summary, the MDR-FC assay provided reliable detection of major β-lactamase families, with some systematic discrepancies for SHV/CMY-related variants. These results indicate that while the assay is suitable as a rapid screening tool, confirmatory WGS remains essential for comprehensive β-lactamase characterization.

### 2.2. Detection of Genes Conferring Resistance to Aminoglycosides, Macrolides, and Sulfonamides

The MDR-FC assay included *aac(6’)-Ib*, *armA*, *rmtB*, and *rmtC* for aminoglycosides; *ermB* for macrolides; and *sul1*, *sul2*, *sul3* for sulfonamides. Compared with WGS (Table 2; Figure 1a,d), overall agreement was moderate for aminoglycosides (sensitivity 0.58, specificity 0.94) and high for macrolides and sulfonamides (sensitivity 0.81–1.00; specificity 0.93–1.00).

**Figure 2:**
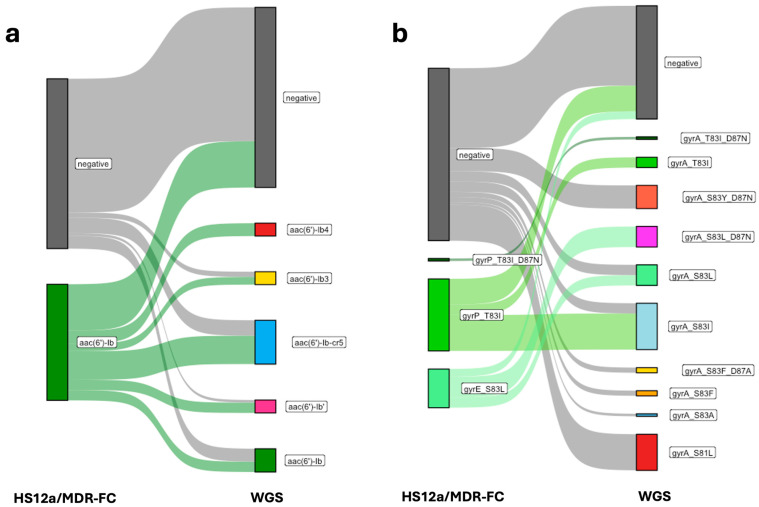
**Comparison of antibiotic resistance gene (ARG) and mutation detection by HS12a/MDR-FC and whole-genome sequencing (WGS).** (**a**) Agreement between HS12a/MDR-FC and WGS in the detection of *aac(6′)-Ib* variants associated with aminoglycoside resistance. Each flow represents the relationship between the ARG detected by HS12a/MDR-FC (**left**) and the corresponding allele identified by WGS (**right**). (**b**) Comparison between HS12a/MDR-FC and WGS for detection of mutations in *gyrA* gene associated with fluoroquinolone resistance. The connections illustrate the correspondence between mutation sites detected by HS12a/MDR-FC and genotypic results obtained by WGS.

For aminoglycosides, the main discrepancies were linked to *aac(6’)-Ib* (SE = 0.66; SP = 0.74; PPV = 0.60; NPV = 0.7; McN test = 0.28, *p* = 0.59), where multiple orthologues (e.g., *aac(6’)-Ib-cr*) complicated detection (Figure 2a) [23,24]. All these variants can have potentially different substrates and diverse resistance profiles. For instance, *aac(6′)-Ib-cr* has gained attention for its ability to confer resistance to both aminoglycosides and fluoroquinolones [25,26]. These variants can evolve and adapt to new antibiotics, posing challenges for treatment options and leading to treatment failures [27,28]. *armA* (SE = 0.43; SP = 0.94; PPV = 0.64; NPV = 0.88; McN test = 21.17, *p* = 0.14) was occasionally under-detected, while *rmtB* (SE = 1.00; SP = 1.00; PPV = 1.00; NPV = 1.00; McN test = Not applicable) was detected with perfect agreement and *rmtC* (SE = 0.50; SP = 0.99; PPV = 0.67; NPV = 0.98; McN test = 0.00, *p* = 1.00) with only minor under-detection.

Macrolides were represented by *ermB*, which was detected with perfect agreement, although the limited number of positives prevents definitive conclusions.

Sulfonamide genes were generally well captured, particularly *sul3* (SE = 1.00; SP = 1.00; PPV = 1.00; NPV =1.00; McN test = Not applicable). The main limitation concerned *sul2* (SE = 0.58; SP = 0.83; PPV = 0.62; NPV = 0.81; McN test = 0.03, *p* = 0.85), which showed frequent under-detection. These results suggest that MDR-FC performs well overall for this antibiotic class; however, some true positives may still be missed, so WGS confirmation remains advisable in selected cases.

### 2.3. Detection of Genes Conferring Resistance to Phenicols and Quinolones

The MDR-FC assay targeted *catB3*, *oqxA*, and *oqxB* for phenicols/quinolones, and *gyrA*, *gyrE-S83L*, *gyrP-T83I*, *parE-S80I*, and their combinations, *qnrA*, *qnrB*, and *qnrS* for quinolones. Compared with WGS (Table 3; Figure 1c,e), overall agreement was moderate, reflecting frequent discrepancies in SNP-based targets.

For phenicols, the main issue concerned *catB3*, which showed poor sensitivity and several false negatives, limiting the assay’s utility for this marker (SE = 0.40; SP = 0.88; PPV = 0.13; NPV = 0.97; McN test = 50.62, *p* = 0.02). In contrast, *oqxA* was detected with relatively high agreement (SE = 0.80; SP = 0.93; PPV = 0.88; NPV = 0.87; McN test = 0.64, *p* = 0.42), while *oqxB* showed less consistent results (SE = 0.55; SP = 0.80; PPV = 0.50; NPV = 0.84; McN test = 0.13, *p* = 0.71), partly explained by the genetic variability within the *oqxB* family (*oqxB13*, *oqxB14*, *oqxB19*, *oqxB20*, *oqxB25*, and *oqxB32*) and the current panel’s coverage.

For quinolones, overall sensitivity was low (0.39; 95% CI 0.31–0.47), despite high specificity (0.95; 95% CI 0.93–0.96). The PPV and NPV were 0.59 (95% CI 0.48–0.69) and 0.89 (95% CI 0.86–0.91), respectively, with a McN test yielding a value of 18.46 (*p* = 1.72). *gyrA* mutations (notably S83L) were consistently captured (SE = 0.78; SP = 0.99; PPV = 0.93; NPV = 0.96; McN test = 0.8, *p* = 0.37), whereas *gyrP-T83I* and *parE-S80I* displayed substantial non-agreement, with a notable number of false positives for *gyrP-T83I*. Discrepancies were mostly associated with FN, and particularly with different SNPs (Figure 2b). For instance, *gyrA*-S83L was under-detected due to the presence of diverse nucleotide sequences that prevented proper probe annealing and resulted in different amino acid substitutions (S83Y, S83L, S83A). Additionally, the MDR-FC assay failed to detect *gyrA*-D87N when co-occurring with S83L variants.

Plasmid-mediated resistance genes *qnrA*, *qnrB*, and *qnrS* were generally detected with good specificity, although under-detection was observed for certain variants. Indeed, for *qnrB* (SE = 0.40; SP = 0.99; PPV = 0.86; NPV = 0.91; McN test = 4.9, *p* = 0.02), WGS identified both *qnrB1* and *qnrB4* variants. Similarly, WGS detected *qnrS1* and *qnrS2* variants, whereas the MDR-FC assay targeted only a general *qnrS* gene (SE = 0.64; SP = 0.97; PPV = 0.84; NPV = 0.90; McN test = 20.83, *p* = 0.14).

Taken together, these findings indicate that while the MDR-FC assay provides useful information for quinolone and phenicol resistance, confirming resistance when detected; however, its limited sensitivity and SNP detection bias prevent it from fully replacing WGS.

## 3. Materials and Methods

### 3.1. Study Setting, Samples, and Genomic Data

**Figure 3:**
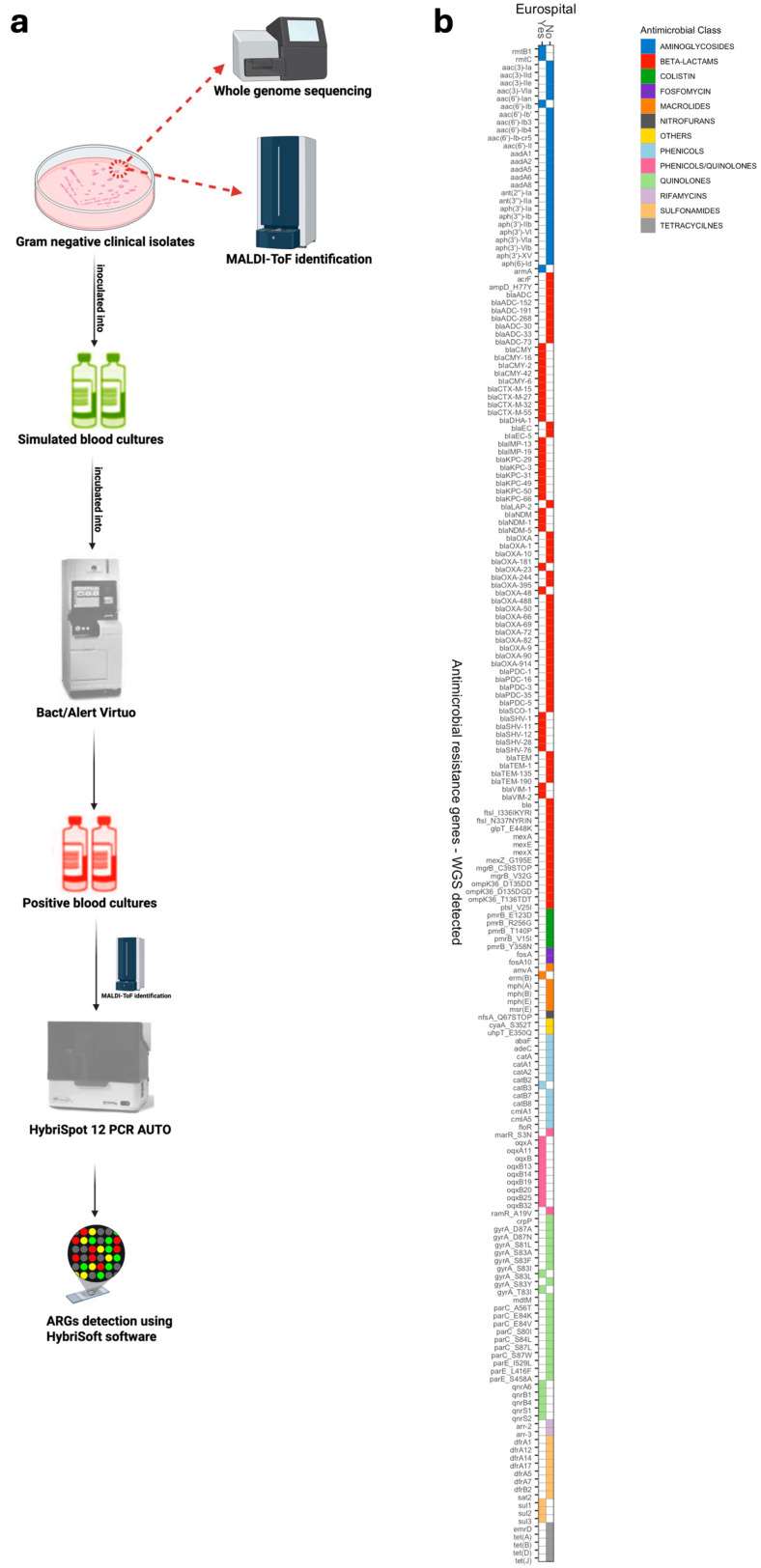
**Schematic flowchart illustrating the evaluation of the HS12a/MDR-FC system and the detection of antimicrobial resistance genes (ARGs).** (**a**) A total of 111 Gram-negative clinical isolates, previously characterized by WGS, were used to inoculate blood culture (BC) bottles (bioMérieux) and incubated in the BacT/Alert Virtuo automated BC system (bioMérieux). Before BC inoculation, each isolate was phenotypically confirmed by MALDI-TOF MS (Bruker Daltonics, Bremen, Germany). Upon positivity, either the aerobic (AE) or anaerobic (ANA) bottle was processed using the HS12a/MDR-FC assay, and results were analyzed with the Hybrisoft software. Each positive sample was also plated according to our standard workflow to exclude contamination or inoculation errors. (**b**) Heatmap comparison of ARGs detected by the MDR-FC assay versus WGS. Genes are grouped by antimicrobial class: aminoglycosides (blue), β-lactams (red), colistin-resistance genes (green), fosfomycin-resistance genes (violet), macrolides (orange), nitrofurans (dark gray), phenicols (sky blue), phenicols/quinolones (purple), quinolones (light green), rifamycins (light violet), sulfonamides (light orange), tetracyclines (light gray), and others (yellow).

This study was conducted at the clinical microbiology laboratory of the Fondazione Policlinico Universitario A. Gemelli IRCCS, a large tertiary-care teaching hospital in Rome (Italy), over a one-year period (April 2023 to March 2024). A set of Gram-negative bacterial isolates (47 *Klebsiella pneumoniae*, 32 *Escherichia coli*, 14 *Acinetobacter baumannii*, 6 *Pseudomonas aeruginosa*, 3 *Enterobacter cloacae* complex, 3 *Proteus mirabilis*, 2 *Citrobacter freundii*, 1 *Citrobacter koseri*, 1 *Providencia stuartii*, 1 *Pseudomonas monteilii*, and 1 *Raoultella ornithinolytica*), previously studied by us [15], were used to obtain PBC samples (one per isolate). Samples were obtained after incubation of BacT/Alert FA or FN blood culture bottles (bioMérieux, Marcy l’Étoile, France) and subsequent positive flagging by the BacT/Alert Virtuo BC automated system (bioMérieux, Marcy l’Étoile, France). The aerobic or anaerobic bottle was analyzed depending on which flagged positive first, as previously described [15]. Information about the isolates is reported in Supplementary Data S1, and the schematic workflow is shown in Figure 3.

Whole-genome sequencing (WGS) experiments for these isolates were originally performed in a previous study [15]. For the present work, raw sequencing data (deposited in the NCBI Sequence Read Archive, BioProject accession number PRJNA1203573) were reanalyzed ad hoc to investigate resistance genes and/or alterations across all major antibiotic classes, including β-lactams, as detailed in our previous work [15,29,30]. Reads were quality-checked and trimmed using the fastp (https://github.com/OpenGene/fastp, accessed on 10 December 2024) and assembled with Unicycler (https://github.com/rrwick/Unicycler, accessed on 10 December 2024). Isolate identity was confirmed using the KmerFinder database (https://bitbucket.org/genomicepidemiology/kmerfinder_db/src/master/, accessed on 10 December 2024). Assembled contigs were screened with AMRFinderPlus (https://github.com/ncbi/amr, accessed on 10 December 2024) and ABRicate (https://github.com/tseemann/abricate, accessed on 10 December 2024), enabling ARG identification. The distribution of ARGs is reported in Supplementary Data S2.

### 3.2. HS12a/MDR-FC Testing

The MDR-FC Kit assay was performed on PBC samples as previously described. Each test contains all reagents required for multiplex PCR amplification on the HybriSpot12 PCR AUTO (HS12a) instrument (Eurospital Diagnostic, Trieste, Italy). The panel includes 55 AMR markers, of which 51 are specific to Gram-negative bacteria (Figure 1b). The set of targets is fixed by the CE-IVD MDR-FC panel (no custom selection by the authors) and reflects manufacturer-curated markers with recognized clinical and epidemiological relevance. Results for aminoglycoside, β-lactam, macrolide, phenicol, quinolone, sulfonamide, and tetracycline resistance genes detected by the MDR-FC Kit are shown in Supplementary Data S3. Briefly, 100 µL of each PBC sample was centrifuged at 4500× *g* for 7 min. The supernatant was discarded, and the bacterial pellet was resuspended in sterile DNase/RNase-free water to a turbidity equivalent to 0.5 McFarland. Three microliters of this suspension were added to 27 µL of DNase/RNase-free water and mixed with the MDR PCR reagent mix. The reaction mixture was then transferred into the cartridge following the manufacturer’s instructions. After amplification, hybridization and colorimetric detection were performed automatically, and the results were analyzed by the Hybrisoft software.

### 3.3. Comparative Data Analysis

The MDR-FC assay was evaluated against WGS for each bacterial isolate used to generate the simulated samples. Only genes included in the commercial panel or their orthologues were considered. Agreement was assessed by comparing, for each gene–species entry, the number of detections reported by MDR-FC with those obtained by WGS. For each entry we classified the outcome as an exact match (MDR-FC = WGS), over-detection (MDR-FC > WGS), or under-detection (MDR-FC < WGS) and computed the exact-match rate as the proportion of entries with identical calls. For readability, we describe agreement as high when the exact-match rate is ≥85%, moderate when 70–84%, and low when <70%; these qualitative labels complement, but do not replace, quantitative metrics.

Results for aminoglycoside, β-lactam, macrolide, phenicol, quinolone, sulfonamide, and tetracycline resistance genes were visualized as chord-chart diagrams, reporting WGS genes (prefix W_), Eurospital genes (prefix E_), and false-positives (FP)/false-negatives (FN). Statistical analyses were performed in R (v4.3.1). Sensitivity (SE), specificity (SP), positive predictive value (PPV), and negative predictive value (NPV) were calculated with 95% confidence intervals (CIs) using the Wilson score interval. McNemar’s test (epiR v2.0.61) was applied to compare discordant proportions. A *p*-value < 0.05 was considered statistically significant.

## 4. Conclusions

The HS12a/MDR-FC system enabled broad and relatively rapid detection of ARGs directly from positive blood cultures, with results available in approximately four hours. Compared with other rapid molecular systems—namely FA-BCID2 (BioFire, Broomfield, CO, USA), eazyplex^®^ BloodScreen GN (Amplex Diagnostics, Gars-Bahnhof, Germany), Molecular Mouse (Alifax, Polverara, Italy), Novodiag^®^ CarbaR+ (Mobidiag/Abbott, Espoo, Finland), and Amplidiag^®^ CarbaR+MCR (Mobidiag/Abbott)—[13,14,15,17], its wider coverage represents a major advantage, although not all targets included in the panel were detected and some discrepancies with WGS, particularly false negatives, were observed. For this reason, WGS was used as the high-standard comparator, given its comprehensive allelic resolution and ability to pinpoint sequence variants underlying discordances.

To our knowledge, this is the first evaluation of the platform directly on positive blood cultures, extending its application beyond bacterial colonies. While the assay cannot replace WGS with comprehensive resistome profiling, it offers a practical option for laboratories where WGS is not routinely available. Integration with complementary methods, such as MALDI-TOF MS, could further enhance diagnostic workflows by combining reliable species identification with rapid ARG profiling. In routine practice, we recommend deploying HS12a/MDR-FC as a rapid screening step with predefined reflex algorithms (e.g., targeted confirmatory tests or WGS) for genes and scenarios at higher risk of under-detection (such as specific *bla*_SHV_ or *bla*_KPC_ variants).

This study has limitations: simulated PBCs may not capture the full complexity of clinical samples (e.g., polymicrobial infections), and the closed-panel design cannot detect off-target or newly emerging alleles; moreover, probe-region polymorphisms can impair hybridization. These constraints can be mitigated by pre-analytical optimization, periodic database updates reflecting local epidemiology and critical SNPs, QC panels including hallmark variants (e.g., SHV G238S/E240K), and reflex WGS for discordant or high-impact cases.

Overall, the HS12a/MDR-FC system shows promise as a molecular AST approach. With further refinement, including expansion of its database to capture novel variants and local epidemiology, it could support faster clinical decision-making, facilitate antimicrobial stewardship, and contribute to improved patient management in diverse hospital settings. From a pragmatic standpoint, the method is potentially cost-effective relative to WGS for early decision support (multi-gene coverage, ~4 h TTR), while definitive, comprehensive characterization remains the domain of WGS.

## Figures and Tables

**Table 1:** **Comparison of HS12a/MDR-FC and WGS results for β-lactam resistance genes.** The symbol “–” indicates that the gene was not detected by either HS12a/MDR-FC or WGS.

Species (No. of Organisms Tested)	No. of Genes Detected by HS12a/MDR-FC (No. of Genes Detected by WGS)											
	*bla*_CMY_	*bla*_CTX-M_	*bla*_DHA_	*bla*_SHV-S_	*bla*_SHV-SK_	*bla*_IMP_-like	*bla*_KPC_	*bla*_NDM_	*bla_OXA-23_*-like	*bla_OXA-48_*-like	*bla*_VIM_	Total Genes
*K. pneumoniae* (47)	14 (8)	17 (14)	4 (4)	0 (46)	–	–	22 (33)	9 (9)	–	9 (7)	1 (2)	76 (123)
*E. coli* (32)	11 (11)	7 (7)	4 (5)	–	4 (4)	–	–	5 (5)	–	5 (2)	5 (5)	41 (39)
*A. baumannii* (14)	–	–	–	–	–	–	–	2 (2)	13 (13)	–	–	15 (15)
*P. aeruginosa* (6)	1 (1)	–	–	–	–	1 (1)		1 (1)	–	0 (1)	2 (2)	5 (6)
*E. cloacae* (3)	2 (1)	–	–	–	–	–	–	–	0 (1)	–	2 (1)	4 (3)
*P. mirabilis* (3)	3 (2)	–	–	–	0 (1)	–	–	1 (0)	-	–	0 (1)	4 (4)
*C. freundii* (2)	2 (0)	–	–	–	–	–	1 (2)	–	–	–	1 (1)	4 (3)
*C. koseri* (1)	–	–	–	–	–	–	1 (2)	–	–	–	–	1 (2)
*P. stuartii* (1)	1 (0)	–	–	–	–	-	–	1 (1)	–	–	–	2 (1)
*P. monteilii* (1)	–	–	–	–	–	–	–	–	–	–	1 (1)	1 (1)
*R. ornithinolytica* (1)	–	–	–	–	–	–	–	–	–	–	1 (1)	1 (1)
Total species (111)	34 (23)	24 (21)	8 (9)	0 (46)	4 (5)	1 (1)	24 (37)	19 (18)	13 (14)	14 (10)	13 (14)	154 (198)

**Table 2:** **Comparison of HS12a/MDR-FC and WGS results for aminoglycoside, macrolide, or sulfonamide resistance genes.** The symbol “–” indicates that the gene was not detected by either HS12a/MDR-FC or WGS.

Species (No. of Organisms Tested)	No. of Genes Detected by HS12a/MDR-FC (No. of Genes Detected by WGS), Conferring Resistance to:								
	Aminoglycosides				Macrolides	Sulfonamides			
	*aac(6’)-Ib*	*armA*	*rmtB*	*rmtC*	*ermB*	*sul1*	*sul2*	*sul3*	Total Genes
*K. pneumoniae* (47)	20 (20)	6 (10)	–	0 (2)	–	32 (40)	12 (14)	1 (1)	71 (87)
*E. coli* (32)	10 (7)	–	1 (1)	2 (2)	2 (2)	20 (20)	8 (8)	2 (2)	45 (42)
*A. baumannii* (14)	3 (4)	7 (8)	–	–	–	10 (11)	9 (9)	–	29 (32)
*P. aeruginosa* (6)	3 (3)	–	–	–	–	6 (5)	2 (1)	–	11 (9)
*E. cloacae* (3)	2 (1)	0 (1)	–	–	–	3 (3)	1 (2)	–	6 (7)
*P. mirabilis* (3)	3 (3)	0 (1)	–	1 (0)	–	3 (3)	2 (2)	–	9 (9)
*C. freundii* (2)	2 (1)	-	–	–	–	1 (1)	–	–	3 (1)
*C. koseri* (1)	–	–	–	–	–	–	–	–	–
*P. stuartii* (1)	–	1 (1)	–	–	–	1 (1)	–	–	2 (2)
*P. monteilii* (1)	1 (1)	–	–	–	–	1 (1)	–	–	2 (2)
*R. ornithinolytica* (1)	1 (1)	–	–	–	–	1 (1)	–	–	2 (2)
Total species (111)	45 (41)	14 (21)	1 (1)	3 (4)	2 (2)	78 (86)	34 (36)	3 (3)	180 (194)

**Table 3:** **Comparison of HS12a/MDR-FC and WGS results for phenicol and/or quinolone resistance genes.** The symbol “–” indicates that the gene was not detected by either HS12a/MDR-FC or WGS.

Species (No. of Organisms Tested)	No. of Genes Detected by HS12a/MDR-FC (No. of Genes Detected by WGS), Conferring Resistance to:											
	Phenicols/Quinolones			Quinolones								
	*catB3*	*oqxA*	*oqxB*	*gyrE-S83L*	*gyrE-S83L-D87N*	*gyrP-T83I*	*gyrP-T83I-D87N*	*parE-S80I*	*qnrA*	*qnrB*	*qnrS*	Total Genes
*K. pneumoniae* (47)	11 (5)	37 (43)	32 (42)	0 (1)	0 (9)	22 (0)	0 (9)	5 (34)	0 (1)	5 (8)	8 (11)	120 (163)
*E. coli* (32)	3 (0)	1 (0)	–	14 (17)	0 (8)	1 (0)	0 (8)	19 (13)	–	1 (6)	10 (11)	49 (63)
*A. baumannii* (14)	–	–	–	–	–	–	–	–	–	–	–	–
*P. aeruginosa* (6)	–	1 (0)	–	1 (0)	0 (1)	5 (5)	1 (1)	2 (0)	–	–	1 (0)	11 (7)
*E. cloacae* (3)	–	0 (1)	0 (1)	–	–	–	–	–	–	–	0 (1)	0 (3)
*P. mirabilis* (3)	–	1 (0)	–	–	–	–	–	–	–	–	–	1 (0)
*C. freundii* (2)	1 (0)	–	–	–	–	–	–	–	–	1 (1)	0 (1)	2 (2)
*C. koseri* (1)	–	–	–	–	–	–	–	–	–	–	–	–
*P. stuartii* (1)	–	–	–	–	–	–	–	–	–	–	–	–
*P. monteilii* (1)	–	–	–	–	–	1 (0)	–	–	–	–	–	1 (0)
*R. ornithinolytica* (1)	–	0 (1)	0 (1)	–	–	–	–	–	–	–	0 (1)	0 (3)
Total species (111)	15 (5)	40 (45)	32 (43)	15 (18)	0 (18)	29 (5)	1 (18)	26 (47)	0 (1)	7 (15)	19 (25)	184 (240)

## Data Availability

Data may be available upon request to the corresponding author.

## Supplementary Materials

The following supporting information can be downloaded at: https://www.mdpi.com/article/10.3390/antibiotics14121221/s1: Supplementary Data S1: Genotypically characterized Gram-negative bacterial isolates included in the study; Supplementary Data S2: Antibiotic resistance genes detected by WGS in the study’s isolates; Supplementary Data S3: Antibiotic resistance genes detected by H12sa/MDR-FC in the study’s PBC samples; Supplementary Data S4: Distribution of mutations in *bla*_SHV_ variants and SNP/indel calls relative to the *E. coli bla*SHV-1 reference sequence.

## Abbreviations

The following abbreviations are used in this manuscript:

AE	Aerobic
AMR	Antimicrobial resistance
ANA	Anaerobic
ARG	Antimicrobial resistance gene
AST	Antimicrobial susceptibility testing
CI	Confidence interval
FA	FilmArray
HS12a	Hybrispot12 PCR Auto
IVDR	In vitro diagnostic regulation
LAMP-GN	Loop-mediated isothermal amplification for Gram-negative bacteria
MALDI-TOF	Matrix-assisted laser desorption ionization-time of flight
McN test	McNemar’s chi-squared test
MDR	Multidrug-resistant
MDR-FC	Multidrug resistance-flow chip
MIC	Minimum inhibitory concentration
MM	Molecular Mouse
MS	Mass spectrometry
NPV	Negative predictive value
PCR	Polymerase
PBC	Positive blood culture
PPV	Positive predictive value
SE	Sensitivity
SNP	Single-nucleotide polymorphism
SP	Specificity
TAT	Turnaround time
WGS	Whole-genome sequencing

## References

[1] GBD 2021 Antimicrobial Resistance Collaborators (2024). Global burden of bacterial antimicrobial resistance 1990–2021: A systematic analysis with forecasts to 2050. Lancet.

[2] Rodríguez-Villodres Á., Galiana-Cabrera A., Torres Fink I., Duran Jiménez R., Cisneros J.M., Lepe J.A. (2023). Evaluation of the MDR Direct Flow Chip Kit for the detection of multiple antimicrobial resistance determinants. Microb. Drug Resist..

[3] Menchinelli G., Squitieri D., Magrì C., De Maio F., D’Inzeo T., Cacaci M., De Angelis G., Sanguinetti M., Posteraro B. (2024). Verification of the Vitek Reveal system for direct antimicrobial susceptibility testing in Gram-negative positive blood cultures. Antibiotics.

[4] European Parliament and Council of the European Union (2017). IVDR: Regulation (EU) 2017/745 of the European Parliament and of the Council of 5 April 2017 on medical devices. Off. J. Eur. Union..

[5] Seymour C.W., Gesten F., Prescott H.C., Friedrich M.E., Iwashyna T.J., Phillips G.S., Lemeshow S., Osborn T., Terry K.M., Levy M.M. (2017). Time to treatment and mortality during mandated emergency care for sepsis. N. Engl. J. Med..

[6] Ferrer R., Martin-Loeches I., Phillips G., Osborn T.M., Townsend S., Dellinger R.P., Artigas A., Schorr C., Levy M.M. (2014). Empiric antibiotic treatment reduces mortality in severe sepsis and septic shock from the first hour: Results from a guideline-based performance improvement program. Crit. Care Med..

[7] Walker T., Dumadag S., Lee C.J., Lee S.H., Bender J.M., Cupo Abbott J., She R.C. (2016). Clinical impact of laboratory implementation of Verigene BC-GN microarray-based assay for detection of Gram-negative bacteria in positive blood cultures. J. Clin. Microbiol..

[8] Castañeda-Barba S., Top E.M., Stalder T. (2024). Plasmids, a molecular cornerstone of antimicrobial resistance in the One Health era. Nat. Rev. Microbiol..

[9] Bennett P.M. (2008). Plasmid encoded antibiotic resistance: Acquisition and transfer of antibiotic resistance genes in bacteria. Br. J. Pharmacol..

[10] Carattoli A. (2013). Plasmids and the spread of resistance. Int. J. Med. Microbiol..

[11] Bush K., Bradford P.A. (2020). Epidemiology of β-lactamase-producing pathogens. Clin. Microbiol. Rev..

[12] Majumder M.A.A., Rahman S., Cohall D., Bharatha A., Singh K., Haque M., Gittens-St Hilaire M. (2020). Antimicrobial stewardship: Fighting antimicrobial resistance and protecting global public health. Infect. Drug Resist..

[13] Mauri C., Consonni A., Briozzo E., Giubbi C., Meroni E., Tonolo S., Luzzaro F. (2023). Microbiological assessment of the FilmArray Blood Culture Identification 2 panel: Potential impact in critically ill patients. Antibiotics.

[14] Bach K., Edel B., Höring S., Bartoničkova L., Glöckner S., Löffler B., Bahrs C., Rödel J. (2022). Performance of the eazyplex® BloodScreen GN as a simple and rapid molecular test for identification of Gram-negative bacteria from positive blood cultures. Eur. J. Clin. Microbiol. Infect. Dis..

[15] Ivagnes V., De Maio F., Baccani I., Antonelli A., Menchinelli G., Rosato R., Cafaro G., Santarelli G., Falletta F., D’Inzeo T. (2025). Detection of β-lactam resistance genes in Gram-negative bacteria from positive blood cultures using a microchip-based molecular assay. Front. Cell. Infect. Microbiol..

[16] Fiori B., D’Inzeo T., Di Florio V., De Maio F., De Angelis G., Giaquinto A., Campana L., Tanzarella E., Tumbarello M., Antonelli M. (2014). Performance of two resin-containing blood culture media in detection of bloodstream infections and in direct matrix-assisted laser desorption ionization-time of flight mass spectrometry (MALDI-TOF MS) broth assays for isolate identification: Clinical comparison of the BacT/Alert Plus and Bactec Plus systems. J. Clin. Microbiol..

[17] Holma T., Antikainen J., Haiko J. (2021). Evaluation of three molecular carbapenemase tests: Eazyplex SuperBug complete B, Novodiag CarbaR+, and Amplidiag CarbaR+MCR. J. Microbiol. Methods..

[18] Anton-Vazquez V., Hine P., Krishna S., Chaplin M., Planche T. (2021). Rapid versus standard antimicrobial susceptibility testing to guide treatment of bloodstream infection. Cochrane Database Syst. Rev..

[19] Andersson P., Harris T., Tong S.Y., Giffard P.M. (2009). Analysis of *bla*_SHV_ codon 238 and 240 allele mixtures using Sybr green high-resolution melting analysis. Antimicrob. Agents Chemother..

[20] Castanheira M., Simner P.J., Bradford P.A. (2021). Extended-spectrum β-lactamases: An update on their characteristics, epidemiology and detection. JAC Antimicrob. Resist..

[21] Liakopoulos A., Mevius D., Ceccarelli D. (2016). A review of SHV extended-spectrum β-lactamases: Neglected yet ubiquitous. Front. Microbiol..

[22] Nüesch-Inderbinen M.T., Kayser F.H., Hächler H. (1997). Survey and molecular genetics of SHV beta-lactamases in Enterobacteriaceae in Switzerland: Two novel enzymes, SHV-11 and SHV-12. Antimicrob. Agents Chemother..

[23] Plattner M., Catelani M., Gmür S.L., Hartmann M., Kiliç F., Haldimann K., Crich D., Hobbie S.N. (2024). Phenotypic differentiation within the *aac(6′)* aminoglycoside resistance gene family suggests a novel subtype IV of contemporary clinical relevance. Antibiotics.

[24] Lin N., Xu W., Huang D., Liu C., Lu J., Zhu M., Bao Q., Pan W. (2025). *aac(6′)*-*Iaq*, a novel aminoglycoside acetyltransferase gene identified from an animal isolate *Brucella intermedia* DW0551. Front. Cell Infect. Microbiol..

[25] Liang S., Cai W., Mao R., Chen M., Dai X., Jin X., Kong W. (2025). Three simple and cost-effective assays for AAC(6′)-Ib-cr enzyme activity. Front. Microbiol..

[26] Gharbi M., Abbas M.A.S., Hamrouni S., Maaroufi A. (2023). First report of *aac(6′)-Ib.* and *aac(6′)-Ib-cr* variant genes associated with mutations in *gyrA* encoded fluoroquinolone resistance in avian *Campylobacter coli* strains collected in Tunisia. Int. J. Mol. Sci..

[27] Park C.H., Robicsek A., Jacoby G.A., Sahm D., Hooper D.C. (2006). Prevalence in the United States of *aac(6′)-Ib-cr* encoding a ciprofloxacin-modifying enzyme. Antimicrob. Agents Chemother..

[28] Ramirez M.S., Nikolaidis N., Tolmasky M.E. (2013). Rise and dissemination of aminoglycoside resistance: The *aac(6′)-Ib.* paradigm. Front. Microbiol..

[29] De Maio F., Bianco D.M., Santarelli G., Rosato R., Monzo F.R., Fiori B., Sanguinetti M., Posteraro B. (2025). Profiling the gut microbiota to assess infection risk in *Klebsiella pneumoniae*-colonized patients. Gut Microbes..

[30] Posteraro B., De Maio F., Motro Y., Menchinelli G., De Lorenzis D., Marano R.B.M., Aljanazreh B., Errico F.M., Massaria G., Spanu T. (2024). In-depth characterization of multidrug-resistant NDM-1 and KPC-3 co-producing *Klebsiella pneumoniae* bloodstream isolates from Italian hospital patients. Microbiol. Spectr..

